# Structured Decision-Making: Using Personalized Medicine to Improve the Value of Cancer Care

**DOI:** 10.3390/jpm3010001

**Published:** 2012-12-27

**Authors:** Bradford R. Hirsch, Amy P. Abernethy

**Affiliations:** 1 Center for Learning Health Care, Duke Clinical Research Institute, PO Box 17969, Durham, NC 27715, USA; E-Mail: Bradford.hirsch@duke.edu; 2 Division of Medical Oncology, Department of Medicine, Duke University Medical Center Box 3436, Durham, NC 27710, USA

**Keywords:** personalized medicine, oncology, comparative effectiveness research, cancer, structured decision-making, time limited trials

## Abstract

Cancer care is often inconsistently delivered with inadequate incorporation of patient values and objective evidence into decision-making. Utilization of time limited trials of care with predefined decision points that are based on iteratively updated best evidence, tools that inform providers about a patient’s experience and values, and known information about a patient’s disease will allow superior matched care to be delivered. Personalized medicine does not merely refer to the incorporation of genetic information into clinical care, it involves utilization of the wide array of data points relevant to care, many of which are readily available at the bedside today. By pushing uptake of personalized matching available today, clinicians can better address the triple aim of improved health, lowers costs, and enhanced patient experience, and we can prepare the health care landscape for the iterative inclusion of progressively more sophisticated information as newer tests and information become available to support the personalized medicine paradigm.

## 1. Introduction

The delivery of medical care is undergoing fundamental change. In no area is this more apparent than in cancer. For decades, the treatment armamentarium available to oncologists remained limited despite substantial progress in other areas. More recently, this paradigm has shifted, with many of the drugs that are newly approved by the United States Food and Drug Administration being used to treat cancer [1]. As an example, five new therapies have been approved to treat prostate cancer over the past five years, many compared only to placebo prior to approval. As such, there is little comparative data about their relative efficacy and the appropriate treatment sequence. Accumulating insights regarding the genomic underpinnings of disease and the relative efficacy of treatments in different patient populations holds great promise to tailor anti-cancer interventions to those most likely to benefit, but fulfillment of this potential remains elusive [2,3,4]. Within this backdrop, it is evident that the value of the care that patients receive can be better defined and optimized if early progress on a few fronts continues to gain momentum. 

Understanding the value of treatments relies first on the generation of more robust data about the care being delivered. New data infrastructure is being developed that will enable change under the American Recovery and Reinvestment Act (ARRA) of 2009 and the Patient Protection and Affordable Care Act (PPACA) of 2010. Meaningful Use requirements included in ARRA are fueling the uptake of electronic health records (EHRs) across the country, which provide more robust data than legacy systems [5]. PPACA has led to a new focus on patient centered care and comparative effectiveness research through the Patient Centered Outcomes Research Institute (PCORI) [6]. Biorepositories are being embraced that allow for the storage and analysis of tissue samples in order to understand the role of genetics and epigenetics within oncology and other diseases [7]. Federated data systems like the Mini-Sentinel Network are being built that allow large-scale aggregation of data [8]. The list of evolving opportunities is long and critically important, as data is the key substrate if we hope to make progress.

To date, gains in care efficiency and personalization from these movements remain limited, but it is still too early to judge their ultimate impact. It is often stated that health care has one of the lowest productivity gains from technology of any industry because of a lack of uptake of new tools and that it can take 17 years for new findings to enter routine clinical practice [9,10]. Driving forces behind this failure include misaligned incentives, a lack of shared understanding between the clinical community and the data scientists, deficiencies in infrastructure (both in care delivery and information technology), and reservations among the clinical community about the role of technology in the health care. A national focus on data, data sharing, biomarkers (of all types), and the integration of technology will start to break down the walls and catalyze progress towards personalized medicine, but it is critical that the clinical and research communities help to guide change instead of leaving it in the hands of industry and policy experts. 

Perspective is important when defining the value of personalized medicine. Patients define health care value in terms of quality of the patient-physician relationship and alignment of care with personal goals [11]. Oncologists and third party payers often focus on objective definitions of effectiveness and efficiency in specific clinical scenarios (e.g., impact of targeted treatment for BRAF V600E mutated melanoma on overall survival). Perhaps, within the personalized medicine community, we are so focused on biomarker driven care that we have missed the opportunity to define the near-term value statement for personalizing care within the context of patient-defined value and patient-centered care. One way to make progress is to demonstrate what has value and what does not by using accessible language and concepts with personal meaning. Outcomes such as progression free survival or time to event are abstract concepts, whereas more patient-centric outcomes like quality of life, ability to walk or run, and symptoms such as pain are more easily understood. 

The role of biomarkers is often focused upon as the heart of personalized medicine. Over the past decade, many new discoveries have been made about the inherent genetic heterogeneity within previously defined groups such as those with lung or colorectal cancer [12]. More recently, even treatments such as aspirin have found increasingly targeted populations such as its use with PIK3CA-mutated colorectal cancer [13]. There are innumerable reviews of the role of various biomarkers such as KRAS in colorectal cancer [14] or BRAF in melanoma [15]. The purpose of this discussion is not to focus on these issues, but instead to show how genomics can be one of many factors integrated into the new model of care delivery in order to improve understanding and outcomes. Instead of waiting for new genomic discoveries, we are working to build the systems that will integrate them quickly into practice and drive new discoveries. 

**Figure 1:**
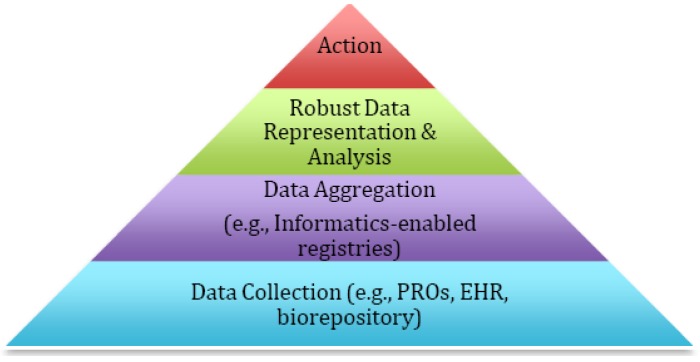
The Requirements for Personalized Medicine.

In this manuscript, we will highlight our experience with personalized medicine in the research and clinical contexts. We will describe how the collection of patient reported information such as patient reported outcomes (PROs) has facilitated the inclusion of the patient voice in care and allowed for personalization in real-time in a manner easily understood by patients and the health care team. We will show how informatics-enabled registries that combine PROs and data from sources such as EHRs and biorepositories allow for the integration of the patient experience and biological factors in order to inform multivariate prediction models and to generate automated, tailored interventions and education. Finally, we highlight how new data can be used to enhance care through time-limited trials (TLTs) that facilitate communication between patients and clinicians. The basic framework that connects the different pieces is shown in Figure 1. Ultimately, new data approaches should be embraced and coordinated in order to better understand the impact of care on a patient’s experience and outcomes, providing new insights into value from both a patient-defined and ultimately societally-defined perspective. The pathways we describe highlight demonstration models of how the largely untold stories of personalized medicine are already moving the field forward using a unique approach that, on the surface, looks exceptionally different from biomarker driven prescription of anti-neoplastic therapies, but, in reality, uses the same principles of matching health care intervention to patient need based upon progressively more tailored real-time data. 

## 2. Data Generation

New data sources are a critical piece of the puzzle. Without them, personalization will remain elusive. As examples, we will highlight how our work with PRO data collection systems and informatics-enabled registries has supported personalization. 

### 2.1. Patient Reported Outcomes

PROs represent the collection of data on a patient’s health status directly from the patient, bypassing the traditional approach of interpretation and documentation by a clinician. Ample evidence shows that collection of PROs avoids misinterpretation by physicians and provides more robust data to be used across clinical care, research, and business operations [16,17,18]. Over the past decade, the Duke Center for Learning Health Care (CLHC) has been one of a handful of organizations to develop a robust PRO capture system, referred to as the electronic patient reported outcomes (ePRO) system [19,20,21]. Our most commonly used PRO instrument is the Patient Care Monitor v2.0 (PCM), because it simultaneously facilitates a clinical review of systems, patient reporting of day-to-day concerns, and research data collection. The PCM consists of 86 questions for women and 80 for men. It has been shown to take an average of 11 min to complete and is both feasible and reliable [20,22,23,24]. We have designed the ePRO platform to be accessible from any internet-enabled device, whether in the clinic (usually by tablet device) or outside the clinic (e.g., tablet, laptop, desktop, smartphone).

The ePRO system has been used at more than 30 academic and community oncology sites in the United States. Drivers of uptake of the ePRO system include financial justifications from improvements in clinical efficiency, a desire for greater patient engagement, and the need for new quality reporting and research capabilities. We have already seen the impact at sites using ePRO. For instance, the prevalence of sexual distress among our cancer patients was fully appreciated for the first time following implementation of the ePRO system at the Duke Cancer Institute. More than 30% of patients with breast, lung and gastrointestinal cancer were found to have moderate to severe sexual distress [25,26,27]. As a result of these findings, new educational and clinical care processes have been put in place and a randomized controlled trial opened to help understand how to address the issue. In another example, cancer pain treatments are being matched to people who need them in real time. Reports generated by the ePRO system highlight patients with pain as they present to the clinic; characteristics of the pain and its severity are documented, and the type and intensity of intervention is matched to the problem. On subsequent clinical encounters, the impact of the treatment choice is monitored using the ePRO system and therapies can then be further adjusted, using iterative cycles of assessment, targeted intervention, and review. 

Many believe that implementation of such a system rests purely on the hardware and software tools involved. In our experience, these are among the easiest hurdles to overcome. One can relatively easily develop a software interface that allows patients to fill out clinical surveys. Much more challenging is the development of valid and reliable survey tools, the process redesign required in clinics to align the use of information systems within the clinical care being delivered, and the requirement for analytic capabilities that convert the raw data into actionable information that clinicians can utilize in real time. These stumbling blocks frequently relegate real-time PRO data collection and use to an “untold story” of personalized medicine, but, when surmounted, result in a clinical care delivery system that is highly patient-centric and directly fosters personalization of care.

### 2.2. Informatics Enabled-Registries

A registry is a collection of patient records assimilating longitudinal information that characterizes and monitors people included in the cohort [28]. Typically registries have pre-specified criteria for patient inclusion, such as all people with a specific disease (e.g., stage IV cancer), exposed to a specific intervention (e.g., spine surgery), or receiving similar health care (e.g., accountable care organization). Outcomes are tracked over time, such as cancer-related survival, change in tumor burden, toxicity of anti-neoplastic therapies, symptoms, quality of life, and health resource utilization. In the case of biospecimens, the primary purpose of a registry can be to provide clinical annotation to document the phenotype that aligns with specific biology; or, the primary purpose of the registry can be focused on the clinical cohort and the biospecimen data can be just one data type among the overall dataset.

Informatics-enabled registries draw from many data sources to automatically aggregate and harmonize data in a centralized data warehouse, allowing far more robust queries and insights than would be possible from any single source. Architecturally, the warehouse can be a single organized database (albeit usually portioned into smaller units called “datamarts”), a loosely laced group of databases accessed when needed to create a virtual warehouse (also known as a “federated” model), or a combination of these models [28,29]. As an example at Duke, strides have been made to aggregate data across the EHR, tumor registry, billing, ePRO and clinical trials systems in order to design a database that can provide deeper insights into critical questions [30]. These registries, once formed, augment all of the systems that they draw from. For instance, the Duke data warehouse acts as clinical annotation system for our biorepository, ensuring that the biological specimens are annotated with continuously aggregating data. Without the registry to bridge the two systems, it would take an overwhelming amount of work to match and analyze clinical data with biological data derived from the biospecimen. Importantly, the pool of data in the electronic data warehouse is continuously aggregating in line with the longitudinal evolution of day-to-day health care that naturally occurs. For example, a patient with newly diagnosed colon cancer might interact with the health care system and have data collected during his annual checkup, appointment with a gastroenterologist, screening colonoscopy, pathology’s review of the biopsy, appointment with an oncologist, and chemotherapy treatment visits. All of the health care data collected during each of these events is tracked in the registry and provides a longitudinal clinical picture that is matched to the cancer tissue samples that were collected. Data can be analyzed at any level of aggregation—institutional, across a network of sites, regional or national, depending upon whether adequate data dictionaries are available and information can flow from one system to the next. 

In order to accomplish this, we have needed to work through a series of complex challenges such as concerns about data quality and the derivation of key variables. As this type of data aggregation represents a new and evolving territory, basic data quality concerns have required the development of systematic solutions. For instance, there is substantial variation in the likelihood that certain data fields are collected. If only the final result of an analysis is reported (*i.e*., 40% of patients are positive for a given mutation) then the nuances of the data are often unknown; in this example, only a small subset of patients may have had the mutation analyzed because known predictors of presence or absence of the mutation make routine biomarker testing unnecessary, thereby skewing the results. As another example of the challenges encountered with large-scale clinical data aggregation, data drawn from an ever-growing number of systems may result in conflicts among the data elements replicated across systems; for example, patient race may be recorded four different ways in eleven different medical data systems and the correct value must be defined. This arises for a number of reasons, including varying definitions of fields between sources and inaccuracies of input entry. Using models to predict the reliability of different sources, an algorithm can be developed to resolve such conflicts, choose the most likely appropriate answer, and add a “tag” to the data element (also known as metadata) indicating the likelihood that the data point is indeed correct. Another interesting and critical issue is the complex challenge of defining and populating critical variables that aren’t readily accessible within systems today, such as tumor response. While many data elements are critical to comparative effectiveness research, they are not always codified and are often buried in radiology reports and clinical notes. Electronic methods to harvest the data, such as prediction algorithms and natural language processing, may or may not be accurate and must be validated. Attempts to add more codified fields and to harness the growing computational power of networks to start sifting through the growing amount of data will help address the gap over time. Meanwhile, clinical chart review and completion of supplementary case report forms can serve as a backup method when needed.

As these complexities are resolved, informatics-enabled registries have great potential to provide critical data needed to support the practice of personalized medicine and personalized health care discoveries. Clinicians interacting with the registry can derive honed understanding of a patient’s risk and likelihood of response. Meanwhile data aggregating in the registry can support the transition of research from a linear to an iterative process. New biological discoveries can be tested in a succession of closely-linked clinical trials from phase I to phase III, put into practice if the findings are promising, and monitored for impact in the general population. Aggregating data from the general health care population then feeds the discovery engine for further innovation. In this way, informatics-enabled registries help to support the concept of learning health care. Collaborative high level stakeholder meetings, such as the Learning Health Summit by the Kaiser Family Foundation [31], Digital Learning Collaborative of the Institutes of Medicine (IOM) [32], and the IOM’s National Cancer Policy Forum are helping to shape a national data and infrastructure roadmap to move the field forward [33,34,35].

## 3. Application

Once data are available, they can be used to support personalized medicine and tailored care while simultaneously augmenting our understanding of the quality, efficacy, and value of the care being delivered. Key features include data use and reuse, integration of the patient voice, and real-time decision-support.

### 3.1. Structured Support

Data collected in real-time can be used to support multiple actions directly intended to tailor care for the individual patient based upon patient-level data. For instance, we are developing the capability to deliver targeted education and structured interventions through the use of the ePRO system. Patients can access the system across settings, whether in the clinic waiting room or at home. Tailored education and interventions can then be provided, based on a variety of inputs including the patient’s diagnosis, clinical findings, and PROs. If a patient documents a concerning progression of pain or meets a predefined threshold for troublesome insomnia, a tailored video is delivered which provides personalized education regarding the symptom, self-management solutions, and directions to additional resources. Education received is documented in aggregating databases in order to meet clinical documentation and billing requirements. Activities occurring during what would previously have been down time for the patient (e.g., sitting in the clinic waiting room), enable increased insights for the patient and allow the provider to spend more time focusing on higher-level issues. We hypothesize that improved patient education can improve outcomes and this is currently being studied. 

Similarly, systems are being developed in which PRO responses, coupled with additional data points, prompt automated interventions from clinical staff. Instead of waiting for a patient’s pain to progress to the point of an unplanned clinic visit or hospitalization, interventions will be proactively triggered. This might consist of a call from a clinician or a visit from a hospice nurse. In another solution being tested, PRO responses are triggering behavioral medicine interventions delivered via video-conferencing on mobile devices to the patients who are most likely to derive benefit (e.g., people with pain scores >3 and positive sense of self efficacy).

### 3.2. Clinical Decision Support

Clinical decision support (CDS) refers to the analysis and presentation of aggregated data to provide guidance to clinicians to tailor care for the individual patient. Numerous studies have outlined the benefits of clinical checklists [36,37,38]. CDS is a logical extension of this approach, meant to help provide reminders and guidance about the latest and most evidence-based care patterns. A key feature is the matching of patient-level data to current best evidence and practices, which can accelerate diffusion of new evidence [9]. A common concern of randomized clinical trials is their limited applicability to routine clinical practice settings [39]. CDS, driven by real time clinical findings, can fundamentally change this paradigm. 

There are innumerable types of CDS systems in development. Let us provide an example of their use to support care pathways and personalized medicine today. Individual patient data can be used to make personalized cancer treatment recommendations such as advising vemurafenib for a person with metastatic melanoma and a BRAF V600E mutation. Now imagine a panel of patients all of whom have melanoma with the BRAF V600E mutation and who receive vemurafenib; their data are aggregating in the informatics-enabled registry. Perhaps a cluster of patients in the panel is noted to develop peripheral neuropathy. A multivariable prediction model is derived using the registry data including information on the sites of disease involvement, lab variations, and genomic markers to identify people at risk of developing the peripheral neuropathy. A cluster of early symptoms serves as an early warning. By inputting a person’s personal characteristics into the multivariable model, a risk score for development of peripheral neuropathy is generated. When the telltale symptom cluster starts to develop in a person at high risk, the vemurafenib is stopped; if the person is low risk, other reasons for the symptom cluster might be sought before dose adjustments to the vemurafenib are made. The ability to use aggregating registries to generate continuously updating prediction models that allow continual refinement of the evidence being used to drive care is an exciting opportunity. While pilot projects must be very carefully controlled to prevent unnecessary risks to patients, we believe that combining these insights with data about the patient’s personal preferences will help us to determine what to do in the context of patient defined goals and values.

Initial systematic reviews have shown the ability of CDS to improve practice patterns but have not yet shown an impact on outcomes [40,41]. This may be largely due to limitations of the available CDS systems studied. CDS is an inevitable part of the future of medical care, so it is in the best interest of clinicians to be involved early and actively in the development process to ensure that, instead of complicating and confining practices, CDS systems provide benefit.

## 4. Time Limited Trials

Another near-term application of the data is through its use in Time Limited Trials (TLTs). TLTs have been gaining recent momentum in which patients and clinicians use a structured process to agree on a management approach [42,43]. As described in a recent manuscript in the *Journal of the American Medical Association*, “a TLT is an agreement between clinicians and a patient/family to use certain medical therapies over a defined period to see if the patient improves or deteriorates according to agreed upon outcomes” [43]. Far too often in oncology, as in other areas of medicine, we choose to continue therapies in an open ended fashion, with no clearly established decision points or acknowledgment about a lack of efficacy. TLTs are an attempt to deal with this divide, and are a critical part of personalization of care at the bedside.

At the outset of therapy, both parties discuss their goals from care. What are the patient’s/ caregiver’s primary values and how can this knowledge be used to direct care? Tools like the ePRO system can help to gather the necessary information to both define goals and document the ongoing balance of risks and benefits. The ePRO system provides an objective tool with which to assess the outcomes that are decided upon such as changes in pain control or functional status. Paired with data from the patient record about trends in lab values (supplied through the informatics-enabled registry), robust systems can be developed to support TLTs and better define the course of care. For instance, a patient with advanced prostate cancer may decide to start docetaxol therapy and agree to a trial of three months. Severe toxicity over that time may prematurely end therapy; agreement at the outset as to his health care goals helps to guide decisions. If a patient’s primary concern is the balance between loss of functional status and the need to shrink the disease, there is a likely a tradeoff he is willing to make. If at the end of the three months, functional status has declined significantly and tumor response is limited as defined by the PSA test and radiographic changes, a discussion of alternative therapies would be appropriate, as previously agreed. Alignment of goals and outcomes empowers the patient. While there is nothing set in stone about the agreement that is reached, it allows patients to be active participants, and leverages the use of data sources to truly support care. Instead of subjectively following functional status, it can now be collected as a data point, explicitly incorporated into the decision-making algorithm and approach to care, and monitored over time.

## 5. Conclusions

The “untold stories about personalized medicine” are exciting. They are about progress on the ground in areas like the collection of PROs, aggregation of data across various sources, and the use of novel data analyses in the provision of care and generation of new evidence. There is angst about the effect of new information technology systems on efficiency and autonomy. The time available in the clinic room is limited. The use of technology to restructure care, improve efficiency, and match the right intervention to the right patient at the right time is critical. This is in direct opposition to the initial introduction of technology into clinics to date, in which it merely replicated the paper chart and negatively impacted clinical flow [44]. But, if done wisely, potential gains to patients and the system through personalization of care far outweigh the drawbacks. Instead of interrupting the clinical flow, discrete data collection systems will allow clinicians to focus on higher-order issues and support personalization of care.

Adoption of the PDSA (plan-do-study-act) cycle framework is an important component of this process. As problems are identified, solutions are chosen that must then be applied, tested and updated in an iterative fashion. Each new analysis is planned within routine clinical care, data is efficiently gathered about the outcomes of the care being delivered, results are studied and then acted on through CDS, guideline updates, and other real-time mechanisms. In this process, the care of patients is continuously updated with the best available evidence. While not a new idea, the PDSA cycle has not been routinely applied, largely due to the lack of an available infrastructure [45]. Instead of always relying on the results of robust trials that can take hundreds of millions of dollars and many years to conduct, insights can now be generated quickly within new data systems and iteratively tested as part of a learning health system [46,47,48]. There are a number of alternative names for PDSA cycles, such as a process recently described in *Annals of Internal Medicine* as design-implement-evaluate-adjust [49], but all have the same principles. It is time that those providing medical care embrace the ability of technology to move the field forward. 

The new approaches we have discussed end in the discussion of value. Value is variably defined depending on the stakeholder. For oncology patients, the value equation may include aspects of care such as the patient experience, response rate to treatments, symptoms, and quality of life. For payers, value may be defined by the balance of cost, utilization of resources, and impact on symptoms/ survival. Many have discussed the notion of value and it remains difficult to define. The greatest potential of the new approaches outlined in the manuscript is in their ability to objectively begin to characterize value on a large scale and enable a far more informed debate about how to optimize and prioritize care. 
